# Newly Graduated Nurses’ Experiences Regarding Job Readiness and Their Development of Professional Authority: A Qualitative Study

**DOI:** 10.1177/23779608251330041

**Published:** 2025-03-21

**Authors:** Heidi Jerpseth, Kari Toverud Jensen

**Affiliations:** 1 158935 Faculty of Health sciences, Oslo Metropolitan University, Oslo, Norway

**Keywords:** qualitative study, newly graduated nurses, work experiences, clinical skills, nursing knowledge, job readiness, professional authority and autonomy

## Abstract

**Introduction:**

The World Health Organization has identified a shortfall of nurses. There is a risk that newly graduated nurses will leave the profession only a few years after completing their education. Little is known regarding the newly graduated nurses’ experiences of practical readiness and development of professional authority during their first year of working.

**Objectives:**

To explore newly graduated nurses’ experiences related to their practical readiness and their development of professional authority.

**Methods:**

Qualitative design. Data was collected from March to April in 2022. The data consist of eight individual interviews with nurses who had been working for almost one year. The data was analysed using Braun and Clarke's reflexive thematic analysis.

**Results:**

Two themes were constructed: Ready or not, and being responsible with or without decision-making authority. The experience of preparedness was related to handling clinical judgement, clinical skills and nursing knowledge. The participants found their role in interdisciplinary cooperation challenging.

**Conclusion:**

The nurses who felt prepared exhibited competence that integrated their personal abilities with clinical judgment, allowing them to apply their skills and knowledge across diverse situations. The participants revealed a discrepancy between perceived responsibility and the level of professional authority that resulted in frustration and a sense of being professionally overshadowed.

## Introduction/Background

In Norway, as in other countries in Western Europe, there is an alarming shortness of nurses. Studies reveal that there is a risk of newly graduated nurses leaving the profession only a few years after completing their education (Hjemsås & Syse, 2023; Parker et al., 2014; Pasila et al., 2017). One issue of concern is related to newly graduated nurses’ expectations of the profession not aligning with reality (Gandi et al., 2011).

Considering the Norwegian context, nurse education is a bachelor's degree regulated by the curriculum regulations for National Regulation Guidelines for Nurse Education (2019) and based on the European Union's Directive 2005/36/EC (European Union, 2005) requirements for academic and clinical practice content, with at least fifty percent (50%) being clinical practice. Although the emphasis on clinical practice is half of the time during nurse education, many newly graduated nurses find the transition challenging. Kramer (1974) studied the experience of transition to professional practice and developed the term *reality shock*. This transition shock is characterised by a myriad of stressors, including heightened stress levels, diminished self-confidence and perceived competence, and suboptimal retention outcomes (Higgins et al., 2010; Kramer et al., 2013). Newly graduated nurses face a particularly vulnerable situation, especially during their first year of professional work. It is widely recognised that nurses frequently experience what is known as *reality shock* (or *transition shock*) when they begin their careers as registered nurses (Ho et al., 2021; Maben et al., 2006; Monaghan, 2015).

It is timely to ask what makes a nurse ready to be a professional, and who decides what it takes to be a professional. The concept of practice readiness has no clear definition, and this might lead to a disparity in nurses’ expectations and support of newly graduated nurses (Harrison et al., 2019). Wolff et al. (2010) claimed that the concept of practice readiness is underdeveloped and warrants a conceptual definition. The underdevelopment of the concept definition is, among other things, related to who has the authority to define the concept. Mirza et al. (2019) conducted a concept analysis of *practice readiness* by reviewing fifteen relevant articles and theses. Their analysis underscored that practice readiness was understood as competence in technical attributes. However, the humanistic qualities that are vital for delivering quality patient care were given limited attention. Practice readiness was found to encompass cognitive, professional and clinical capabilities. However, the inclusion criteria for the concept analysis (Mirza et al., 2019) did not specify who is responsible for defining the concept. The findings from the included articles revealed that the responsibility for defining readiness varied among nurses, nursing students and leaders. Masso et al. (2022) conducted a scoping review of reviews to understand what is known about the practice readiness of newly graduated nurses. Their findings showed that the transition is shaped by many factors, with reality shock being one of the influences on the preparedness of new nurses.

A competent nurse needs to have professional autonomy, which Skår (2009) described as the authority to make decisions and act based on one's professional knowledge. Professional autonomy in nursing is described as multidimensional, applied to the profession or individual nurses (Pursio et al., 2021), in meeting with other health professions but also through handling their own responsibilities as nurses. Pursio et al. (2021) referred to aspects of professional autonomy in nursing as independence in decision-making and the capability to make use of one's own competence. Not only were themes such as shared leadership and healthy work environment considered important, but inter-professional and intra-professional collaboration were also identified as crucial factors. They also claimed that nurses cannot be autonomous if they do not have enough authority.

In this study, we will focus on the newly graduated nurses’ experiences of the transition process in light of practical readiness and development of professional authority during their first year of working.

## Review of Literature

There is extensive research on the transition process for the newly graduated nurses. Based on Kramer's (1974) work, Duchscher (2008, 2009) further developed the term into a transition framework and programme for newly graduated nurses. The research that has been conducted regarding transition is still relevant and, in a study from 2024, Cusack et al. (2024) conducted an online survey to explore the influence of the transition programme on newly graduated nurses. They found that participants described challenges with regard to responsibilities, being involved in clinical incidents and the burden of working in shift. Ortiz (2016) studied professional confidence and how it developed among new graduate nurses during their first year. The study revealed that, in their first year of working, the nurses needed to experience both negative and positive events in order to take care of the patient and handle critical situations. Another study (ten Hoeve et al., 2018) found that novice nurses experience many challenges, such as lack of support from supervisors and physicians, and negative perception of their own competence. Notably, a systematic review identified prominent themes, such as knowledge deficits, the overwhelming nature of clinical work and the pivotal role of workplace support, along with the profound significance attributed to the identity of ‘being a nurse’ (See et al., 2023). Furthermore, Harrison et al. (2019) described four domains of readiness: personal readiness, clinical readiness, professional readiness and industry readiness. Personal readiness influences practice readiness and illustrates the need for graduate nurses to be able to cope with change and manage their care in a dynamic, complex healthcare setting. Clinical readiness describes a level of clinical capability and basic level of nursing care. Professional readiness encompasses the capabilities that graduate nurses require to work efficiently and provide care and includes critical thinking and problem solving. Industry readiness refers to the knowledge and understanding of the system, location, community and organisational regulations (Harrison et al., 2019). Harrison et al. (2019) explored the concept of readiness for clinical work from the perspective of experienced healthcare professionals. However, this study is particularly interested in the experiences of recent graduates. Newly graduated nurses report a disconnect in the clinical knowledge learned in the nurse education and the demands of clinical practice, thus leading to a lack of professional confidence (Henderson & Eaton, 2013).

The focus of this study is therefore twofold by exploring the newly graduated nurses’ experiences of readiness and their perceived professional authority, as a stage of their development of professional autonomy. The aim of the study is to explore newly graduated nurses’ experiences related to their practical readiness, and their development of professional authority, in their first year of working.

## Methods

### Design

This study is part of a larger research project in which we interviewed the same participants over a period of four years. We conducted the first study when the nursing students were in their third year of the bachelor's education programme (Jensen & Jerpseth, 2023). In the current study, we interviewed the same participants one year after they had graduated. We adopted a qualitative, exploratory approach, utilizing individual interviews to investigate the participants’ experiences in alignment with the study's objectives. Our methodology was grounded in a phenomenological and hermeneutical framework. Phenomenology provided insights into the lived experiences of newly graduated nurses, while hermeneutics focused on interpreting the meaning of those experiences (Brinkmann & Kvale, 2015).

The analytic process involved using reflexive thematic analysis, with a six-phase model (Braun & Clarke, 2022). The reflexive thematic analysis is based on an understanding that the participants’ interpretations and meaning indicate their practical readiness and the development of their professional authority.

### Study Setting, Sampling and Data Collection

In the first study, we sent an open invitation via the Canvas learning management system to recruit the participants, and we invited all undergrade nursing students in their third year of their bachelor's degree programme to participate. The sole inclusion criterion was that the students were enrolled in the third year of their programme. In a Norwegian context, the university is relatively large, with nearly 250 students in their third year. However, only nine students agreed to participate in this study. This low response rate may be attributed to the impact of the COVID-19 pandemic (Jensen & Jerpseth, 2023). As the participants had already been recruited as part of the larger qualitative study, we e-mailed the same participants and asked whether they were still interested. Eight of the nine participants agreed to participate. All the participants had worked as a nurse for nearly one year when the interviews took place.

As the participants worked and lived all over the country, we found it most convenient to use the cloud-based video app Zoom, which was connected to Services for Sensitive Data to record interviews (University of Oslo, 2022). To update the demographic data from the earlier interview, we asked the participants about their ages and current workplaces, which were categorised into either primary care or secondary care (see Table 1). The data was collected between March and April in 2022.

**Table 1.** Characteristics of the Participants.

Participant	1	2	3	4	5	6	7	8
Male/Female	F	F	F	F	M	F	F	F
Age (years)	22	28	25	22	29	29	36	32
Workplace^a^	PC	SC	SC	SC	PC	PC	PC	SC
E/V^b^	−	E	E	−	E	E	V	V

a PC = primary care, SC = secondary care ^b^E = prior graduate qualification (e.g., economics, marketing, literary studies), V = prior vocational professions

The data collection consisted of individual semi-structured interviews based on an interview guide (see Table 2). The interview guide was based on previous research (see the introduction and review of literature paragraphs) and our experiences. The interview guide was not validated. We endeavoured to ask questions that would encourage the participants to share their experiences and perceptions from their first years as a newly graduated nurse. The interviews lasted from 30 to 45 min. Both authors took part in all the interviews. The interviews were transcribed verbatim by the last author.

**Table 2.** Interview Guide.

How did you experience the transition from student to nurse? How were you received when you started as a nurse? (Requirements, roles, responsibilities and knowledge) Can you describe a ‘typical day’ at work? Now, after almost a year as a nurse, do you find that the education you have had is relevant to the job you do as a nurse?

### Data Analysis

We used the six-phase reflexive thematic analytic process developed by Braun and Clarke (2022). The six phases are characterised as: (1) familiarisation with the data, (2) coding, (3) generating initial themes, (4) developing and reviewing themes, (5) refining, defining and naming themes, and (6) writing up (Braun & Clarke, 2022). As recommended, we separately listened to the interviews and comments in the transcript text to familiarise ourselves with the dataset. As there were two authors, we organised meetings where we could present our first impressions of the dataset. Some of the impressions were shared, but through this reflexive process, we tried to avoid predefining the themes before coding. In the next phase (2), we made systematic and detailed extracts of the meaning from the dataset into a matrix, which we used to systematise the data material throughout the analytic process. We constructed thematic codes for the eight datasets and searched for patterns or themes. In the third phase, we decided which codes fitted together as patterns of shared meaning and constructed the themes. We also used the stated aim as a guide to ensure that we stayed in line with the intentions of the study. In the fourth phase, we discussed the initial themes and modified some of them. In the next phase (5), we re-read the transcript to check the themes and quotations and how well they worked, both together and individually. Although we followed the structure of these phases, the reflexive thematic analysis is not precisely delineated as we went back and forth in the data material (see Table 3). Both authors agreed on the final themes that described the findings.

**Table 3.** Examples of Analysis.

Meaningful unit	Coding	Generating initial themes	Naming themes
*I came across such a cartoon … who was a nurse and had a site … that as a newly qualified nurse, you often feel that the bachelor's degree you have feels a bit like a weekend course when you first start, and that's probably how it felt that you’d actually learned a lot from this weekend course, but ultimately, it's the year once you’ve started as a nurse when you probably learn the most. (4)*	New role from student to graduate nurse	Feeling prepared or unprepared for the new role	**Ready or not**
*I think what has become a bit special lately is what the education actually says, as you hear the teachers talk about this being challenging. You are going to be tired. Although you hear all that, it doesn’t convey how bad it actually is out there, like in this job and how tired you get. (5)*	Distance between ideal and reality		
*It's always us who do most of the procedures and often set up wound procedures before a doctor has had a look at it, just to get a temporary treatment then before an assessment has been made by the doctor. (8)*	Responsibility with or without decision-making	Being responsible with or without decision-making authority	**Being responsible with or without decision-making authority**
*One thing I really like about this job is that you are never alone as a nurse. (1)* *When you come to work as a nurse, and if you are the only nurse, you always have a responsibility. And you come in with medicines and then they* (patients’) *have questions, and because you only work with extra shifts, that they can’t answer these things … You stand with everything, and you just don’t have the time or the capacity to always be present then. (2)* *… .even if you have colleagues around you, you are in a way alone. It is just expected that you are now a nurse, so you can fix this. No one asks if I know things. It's just assumed as a matter of course. (7)*	A range from never working alone to having the responsibility alone		

### Ethical Considerations

The Norwegian Centre for Research Data approved this study (project no. 845886). The participants had already received an e-mail providing information about the study, and written consent was provided for all three planned studies. However, we sent them an e-mail to ensure that they still wanted to participate. In this e-mail, we re-informed them about their rights to withdraw from the study at any time in the research process before the analysis. The participants agreed to the use of a videotelephony software program, Zoom, to collect the data. We used the app connected to Services for Sensitive Research data* to record the interviews. The sound files were password protected, and only the researchers were allowed to access the files. The data was de-constructed and stored in accordance with the current guidelines at the University and in accordance with the General Data Protection Regulation (GDPR).

### Rigor and Reflexivity

For this study, individual interviews were used as the data collection method. This involved discussion regarding our preunderstanding and understanding of what was presented by the participants. Throughout the research process, we interrogated the data transcriptions to be able to see different nuances in the text. However, the findings from the first study might have coloured the questions that we asked and our interpretations of the data. We have tried to remain critically reflexive throughout the whole process (Braun & Clarke, 2022). The transparency of the analysis process is obtained by the descriptions of the whole method and process, as shown in Table 3. The dependability was enhanced by presenting the quotes. We followed the (EQUATOR), network reporting guidelines using Consolidated criteria for reporting qualitative research (COREQ) to improve the reliability and value of this study (Tong et al., 2007).

## Findings

The participants were newly graduated nurses working in either primary care or secondary care. The participants’ ages might seem high for newly graduated nurses, but four of them had already completed a graduate qualification and two had previous vocational professions (see Table 1).

The findings revealed several challenges faced by the newly graduated nurses during their transition into practice as a nurse. None of the participants had been offered or joined a graduate nurse transition programme. Some of the newly graduated nurses experienced that their educational preparation provided the necessary skills to practice safely, whilst others were not satisfied with their educational preparedness, especially related to technical skills and capacity to navigate in the healthcare hierarchy. The participants experienced uncertainty related to their own role of being a nurse, especially considering how they defined their responsibility and authority. They felt a disconnect between assuming responsibility as a nurse and having the authority to make decisions. Two themes were constructed: (1) Ready or not, and (2) Being responsible with or without decision-making authority.

### Ready or not

The participants experienced the transition from nurse education to practicing as a nurse differently. Some of them stated that they were prepared because they perceived that what they had learned throughout their bachelor's programme gave them enough competence to be able to work as a professional nurse. This competence included their abilities and clinical judgement that enabled them to adapt their knowledge and skills in different circumstances. This competence also made the nurses more self-assured and confident that they could cope with the challenges that they encountered in practice.

First, I became extremely stressed because I had never received a patient with newly diagnosed diabetes before. Then, I sat down. I proceeded to retrieve the procedure on how this should be done, and it was a very well-detailed procedure, with all the necessary information. I located the insulin that needed to be administered, as well as the blood sugar monitoring equipment and everything else. Afterwards, I went in to see the patient. I introduced myself and informed (the patient) that I would be assisting from now on, and this is what will happen in the next few hours. I also mentioned that there would be a lot of new information to absorb, but that it was crucial that we start somewhere, and we would begin by checking the blood sugar. I explained in detail how we would go about everything. (3)

However, some of the participants experienced that they lacked the skills that they deemed to be important to being able to work as a nurse. They experienced that they had incomplete operational competence, and this was especially related to their lack of proficiency in procedures upon entering the workforce, coupled with being unprepared in encounters with the realities of workplaces.

The skills I was missing, like clinical procedures, made me feel quite insecure at that time. Figuring out how to learn them and identifying the necessary knowledge was a challenge for me. (3)

Another area that posed significant challenges was the workplaces’ hierarchy and organisational dynamics. Participants perceived the transition to the professional world as challenging, particularly due to their lack of preparedness for the hierarchical culture among other professions. The nurses experienced that their professional voice was not taken as seriously as they had expected, especially related to their cooperation with the physicians.

It makes such a big difference knowing that the physicians you work with are regular and familiar faces on the ward. Half of the job is knowing what ‘that physician’ tends to forget – for instance, always ordering chemotherapy. If ‘he’ is performing surgery, I need to be ready to order it (chemotherapy), or else the patient won’t receive it. It's like being an on-call babysitter. I wasn’t quite prepared for being a babysitter and just following orders. I really enjoy discussing and finding solutions, arriving at a common decision. Reflecting together on what's right and what should be done. (2)

Some of the participants experienced that being together with nurses that were newly graduated gave them a kind of security, a feeling of inclusion in the community. However, some of them felt that they were extremely alone and had little support and guidance during their transition into being a nurse. They had a rather uneasy feeling when starting as a nurse in a new job.

Even with colleagues nearby, there's a sense of solitude. The expectation is that, as a nurse, you must just handle it. Nobody inquiries about your ability to handle it – it's simply taken for granted. (6)

The nurses’ sense of being unprepared was further fuelled by their experiences of being overworked. They worked under pressure that made them feel it was impossible to care for the patients in the way that they had learned and wanted to perform. They claimed that their education did not prepare them for the reality and instead presented a glossy picture of the everyday life of a nurse.

Being well-prepared for the realities of everyday life as a nurse is crucial. The idealised image of a perfect nurse often doesn’t capture the time and dedication required for providing quality care. Nursing indeed takes time, and it's essential to allow oneself the necessary time for each patient. But then you must go to the next patient. (7)

The findings revealed participants’ nuanced experiences of being ready for their professional work. This is evident in how they perceived their own competencies related to skills, procedures, knowledge and the hierarchical culture of their workplaces. The participants also highlighted the importance of being included in the community of practice and having their professional opinions heard. This can be understood as significant in addressing their feelings of loneliness and lack of professional participation, as some of them described.

### Being Responsible with or Without Decision-Making Authority

The nurses were aware of their duty to be responsible. They accepted and described their own willingness to be in charge. Several participants experienced that they shouldered responsibilities. They also noted that their input played a significant role in the decision-making processes pertaining to patient care. Despite this, some of the participants experienced uncertainty related to their own role as a nurse, especially considering how they defined and demarcated their responsibility. They felt a disconnect between assuming responsibility as a nurse and having the authority to make decisions.

… it has probably become even clearer to me that responsibility … We have a lot of responsibility after all … I feel it very much now, that you sometimes have the last word (in a decision) when someone is discharged. (1)

However, it seemed that being responsible did not determine the authority accompanying the position as a nurse. The lack of authority in these experiences was linked to what they perceived as a lack of interdisciplinary cooperation and discussions with the physicians. The nurses experienced that, rather than being involved in the decision-making process, they experienced an absence of interdisciplinary cooperation. This lack of involvement in planning for the patients’ care and treatment made the nurses unsure of their own roles regarding their professional responsibility. They experienced that the nurses’ perspective of caring for the patients was not taken seriously when it came to decision-making regarding the patients’ treatment and time for discharge.

Sometimes, I have to discharge a patient whom I believe is not ready to go home. I am required to prepare the discharge based on the physician's decision, even though I was not involved in that decision-making process. Unfortunately, it is often the patients without a strong voice who end up being ‘pushed aside’. (2)

The participants experienced some of the same lack of authority in the allocation of patients between different institutions and care levels. The nurses described this as frustrating, with a feeling of not doing the best for their patients.

… it is often ‘the municipality’ that decides on the places, so when they think that the patient needs to be discharged (from the hospital) or move on or whatever, it usually happens that way. Yes, it often feels like it is a case of putting money before people … I get frustrated because I feel that I don’t get to do a good job, no matter what I do with the patient; it doesn’t help if they are sent home too early and end up back in the same cycle once more. (7)

The participants encountered a discrepancy between their perceived responsibility and the level of professional authority they believed they should possess. This dissonance resulted in feelings of frustration and a sense of being professionally overshadowed.

## Discussion

The findings highlight that the transition from student to newly graduated nurse is complicated and demanding. However, the participants in this study gave a nuanced picture. Some of the participants felt that their educational preparedness for practice was not satisfactory, especially in relation to their technical skills. This is in line with other studies that show that newly graduated nurses struggling with preparedness for clinical practice (Masso et al., 2022; Milton-Wildey et al., 2014; Missen et al., 2016; Ralph et al., 2014). Other participants felt that their knowledge and experiences were good enough for handling the complex healthcare setting. These experiences might be linked to the concept of personal readiness, which includes the ability to manage relationships and the unpredictable changes that are common in healthcare contexts (Harrison et al., 2019).

Our findings reveal that some of the nurses felt that they were extremely alone in their position as a nurse. They were not offered any graduate nurse transition programme, neither formal nor informal. Transition programmes do have a positive impact on newly graduated nurse retention (Rush et al., 2012). The development of self- and professional confidence, coupled with critical thinking, is essential in order to provide quality in patient care and practice readiness (Bjørk et al., 2014). One might question whether the nurse education sufficiently accounts for the development of professional self-confidence. Ortiz (2016) reports that this confidence developed over the first 12 months and relies on how the newly graduated nurses are treated in their first clinical practice. In addition, a study by Monaghan (2015) revealed that newly qualified nurses experience a lack of confidence in their own abilities, and that they experienced an allocation of too little time to the strengthening of clinical skills. Like other professions, newly graduated nurses need time to become confident in their position as practicing nurses. Is it a misunderstanding to assume that newly graduated nurses, who have spent half of their education in clinical practice, should be more prepared for working life than other recent graduates?

It seems that the transition from being a student to a practicing nurse is more complex and requires different mechanisms than those needed during clinical practice as a student. If that is the case, how can we better understand this phenomenon and prepare students during their education to meet these expectations and requirements? Studies have reported on the complete absence of documentation or agreement on the quantity, quality or type of clinical practice and experiences that are needed to train a skilled and competent nurse (Cipher et al., 2021; Henriksen et al., 2020). One answer might be that nurse education needs to provide greater emphasis on clinical research, reflection skills and theoretical knowledge to strengthen and prepare nursing students for meeting clinical practice as nurses.

Some of the participants were concerned about their lack of technical skills, which they believed were crucial to defining themselves as nurses. This lack of competence is easily noticed and often judged as a deficiency. Although these may seem like small factors that make the difference in becoming a nurse, the participants experienced them as painful and stressful. Skår (2009) described the core components of autonomy as being knowledgeable and confident, in the sense of knowing the patient and knowing what to do, but also daring to meet the unknown situations and to have an overall (holistic) view of the situation and organisation of the care.

Some of the participants in this study experienced a disconnect between assuming responsibility as a nurse and possessing the authority to make decisions. This might be linked to the participants’ lack of self-confidence and professional authority. However, it might also be a relevant question to ask how the nurse education has prepared these new nurses in terms of building professional authority and the extent to which the working environment supports them. This aligns with a study that highlighted a gap between the education nurses receive for communicating with physicians and how these interactions manifest in practice (Forbes & Evans, 2021). Freidson (2001), on the other hand, claimed that *the degree and scope of the authority of a discipline depend on the concrete historical circumstances surrounding its position – its relationship to other disciplines in the social division of labor and to the spirit of the times (p. 158)*. This historical culture of professional hierarchy could still potentially be one of the reasons why newly graduated nurses feel both uncertain and professionally overshadowed. Some of the participants expressed a feeling of not being taken seriously in their work, and cited experiences of being reduced to the role of ‘a babysitter’ and merely ‘following orders’. Being new to a profession and trying to assimilate into the ward's established working culture can be both challenging and frustrating. This might be understood as what Harrison et al. (2019) described as a lack of industry readiness. Participants also reported feelings of being overshadowed in decision-making processes concerning patient discharges, both from physicians and the system (municipal). This is in line with ten Hoeve et al. (2018), who found that novice nurses often experience missed support from physicians and arrogant behaviour. This might partly be understood by how nurses and physicians are trained to emphasise different aspects of clinical reasoning in relation to a patient's needs. Vreugdenhil et al. (2023) conducted a systematic review to explore how nurses and physicians approach clinical reasoning. The physicians had a narrower focus on the patient's illness and its causes; nurses, on the other hand, had a broader focus that encompassed their domain of care. This might be one of the reasons why physicians and nurses sometimes struggle to find a common language to articulate the patients’ needs and what appears to be in the patients’ best interest. In addition, Skirbekk et al. (2018) highlighted that premature patient discharge can be frustrating for both physicians and nurses, and that many clinicians felt that pressure from management to ‘keep patients moving’ was due to budget issues. They often found this approach wasteful and irritating.

The hierarchical culture within the health care system is well known and fosters the maintenance of traditional roles for physicians and nurses. In this culture, it might be difficult for newly graduated nurses to consider themselves as sufficiently experienced to stand up for their patients and to be a participant when it comes to decision-making regarding the patients’ wellbeing (Jerpseth et al., 2017; Thomas et al., 2003). This demands courage and professional authority, together with a distinct nursing voice. Hossain and Clatty (2021) claims that when nurses compromise on basic nursing care, this might cause moral distress. It is well known that experiences of moral distress lead to burnout, as well as nurses leaving the nursing profession (Sheppard et al., 2022).

It takes time to develop a professional authority and a culture that appreciates it. In addition, there is a growing expectation that nurses should be able to combine various sources of information and incorporate these into their decision-making and nursing practice. Thorne (2023, p. 1) has claimed that attempts to define nursing are misguided. She argues the following:

As every nurse knows, the essence of good nursing extends far beyond the “task functions” – the clearing of airways, insertion of a urinary catheter, the cleaning of wound dressings, and so on. What constitutes nursing practice excellence is the application of knowledge and interpretive skills, integrated within the reasoning, judgment, and relational activity that accompanies the enactment of those task functions, through which nurses come to understand the complex process of seeking health and supporting healing as it is enacted in an infinite diversity of human identities, contexts, and situations.

Accommodating this extensive competence takes time – perhaps several years as an executive nurse. As educators, we need to remind the new nurses of this, when they are nearing the end of their bachelor's studies in nursing.

### Strengths and Limitations of the Work

As this study is part of a larger research project, the participants have been interviewed twice. This might be a limitation, as our preconception might be coloured by the first interviews with the participants. In our sample, there was only one male participant. This may have limited the representation of experiences from both genders. On the other hand, only ten percent (10%) of nursing students are men in a Norwegian context, which suggests that the findings still provide a representative picture of the experiences. Additionally, we reflected on our own choices throughout the whole research process by asking ourselves questions, such as ‘How do we know what we know?’ and ‘Could this knowing be understood in another way?’ Another limitation is that we have used a small sample, and the participants might have a special interest in this subject. However, as this is qualitative research, the main focus of interest is on these participants’ experiences. Both researchers are experienced in conducting qualitative research.

### Recommendations for Further Research

Based on this study, there is a need for more research regarding how to strengthen the argumentative knowledge of nursing students and newly graduated nurses. This could be incorporated into nurse education. It seems important to strengthen the students’ ability to advocate for the rights of patients and themselves to be involved in decision-making processes. This future research could take the form of a simulation intervention, where the nursing students are part of an interprofessional project that emphasises argumentative communication and teamwork. Another possibility to gain deeper insights into this field is to conduct longitudinal research where the same nurses will be interviewed three years after graduation. This approach would allow tracking the professional development of nurses beyond their first year of practice and provide valuable insights into how their perceptions of readiness, authority and competence evolve over time.

### Implications for the Profession

Considering the findings from this study, there appears to be a need for more knowledge to ensure that nurses remain in both primary and secondary care. This study is relevant for practice, particularly for leaders in healthcare services. These leaders need to understand the experiences and challenges faced by newly graduated nurses as they transition into their roles. Another potential implication could be that newly graduated nurses receive support to enhance their argumentative skills in professional communication. Leaders need to be aware of their responsibilities to nurture and support nurses, thereby fostering a culture in which interprofessional collaboration and professional dialogue are encouraged. This study is of great significance for nurse educators because it underscores the need to train nursing students in professional argumentation skills for effective interaction with other professional groups. Engaging newly graduated nurses and nursing leaders in dialogues is crucial. The exchange of experiences, particularly those related to being new. Leaders can also provide guidance on how to effectively support roles and foster argumentative communication. This process will encourage nurses to express their viewpoints confidently and constructively, significantly enhancing their professional growth and the overall healthcare environment.

## Conclusion

The study has sought to explore the experiences of newly graduated nurses in relation to their practical readiness and the development of their professional authority during their first year of work. It revealed that these nurses grappled with various challenges throughout their transition, such as understanding their role as a nurse considering their responsibilities and authority. The participants in the study had nuanced experiences of feeling ready – or not – for their professional duties. Those who felt ready demonstrated a competence that encompassed both their personal abilities and their capability to make clinical judgements, which enabled them to apply their skills and knowledge in various situations they encountered. The study also revealed a discrepancy between the participants’ perceived responsibilities and the level of professional authority they believed they should have, which resulted in feelings of frustration and a sense of being professionally overshadowed.
